# Beneficial Effect of Intestinal Fermentation of Natural Polysaccharides

**DOI:** 10.3390/nu10081055

**Published:** 2018-08-09

**Authors:** Tiehua Zhang, Yang Yang, Yuan Liang, Xu Jiao, Changhui Zhao

**Affiliations:** College of Food Science and Engineering, Jilin University, Changchun 130062, Jilin, China; zhangth@jlu.edu.cn (T.Z.); yangyangat1118@163.com (Y.Y.); liangyuan1995ly@163.com (Y.L.); jiaoxu9915@mails.jlu.edu.cn (X.J.)

**Keywords:** polysaccharide, microbiota, dietary fiber, metabolic syndrome, diabetes

## Abstract

With the rapid development of modern society, many chronic diseases are increasing including diabetes, obesity, cardiovascular diseases, etc., which further cause an increased death rate worldwide. A high caloric diet with reduced natural polysaccharides, typically indigestible polysaccharides, is considered a health risk factor. With solid evidence accumulating that indigestible polysaccharides can effectively prevent and/or ameliorate symptoms of many chronic diseases, we give a narrative review of many natural polysaccharides extracted from various food resources which mainly contribute their health beneficial functions via intestinal fermentation.

## 1. Introduction

**Figure 1:**
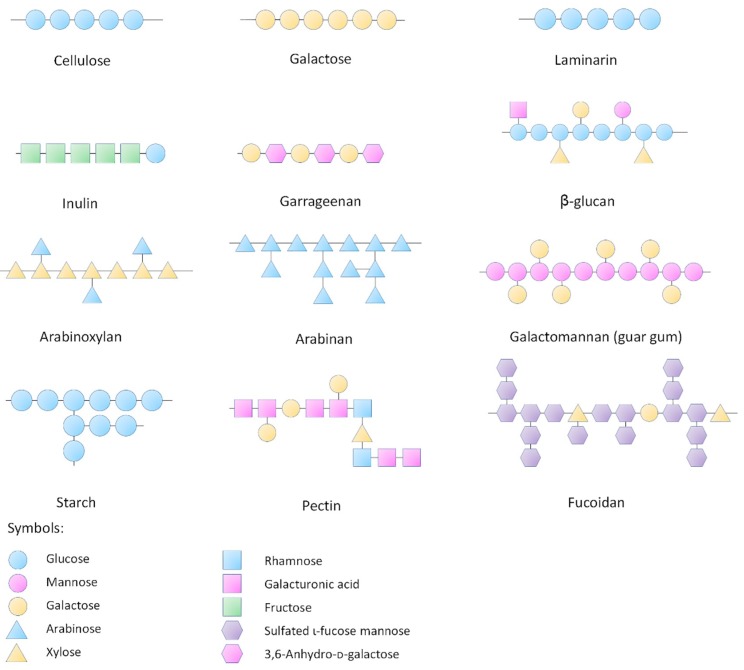
Structures of representative polysaccharides.

Polysaccharides are a class of polymeric molecules composed of long chains of monosaccharide units bound together by glyosidic linkages, which are widely distributed in nature (Figure 1). Many natural products as foods contain a great number of polysaccharides that cannot be completely digested by our digestive system. These indigestible polysaccharides are often called dietary fiber. The typical dietary fiber includes cellulose, hemicellulose, β-glucan, pectin, mucilage, gums and lignin. With advancement of the extraction and identification techniques, many newly found polysaccharides are continuously discovered from various resources. Most of these polysaccharides are considered resistant to digestion in our alimentary system including the resistant starch—a starch fraction that is mainly fermented by the large intestinal microbiota. It needs to be noted that even the digestible starch is also partly fermented by the gut microbiota. Therefore, the focus of this review is the polysaccharides which confer the beneficial effect to health mainly through their fermentability in the gut as well as the physical and chemical properties including water-holding capacity and bile acid binding ability.

## 2. Intestinal Microbiota

Intestinal microbiota is considered a dynamic organ that plays an essential role in maintaining health. The intestinal microbiota is an intricate collection of microscopic organisms in the gut, including more than 100 trillion microorganisms, such as viruses, bacteria, protozoa and fungi [1]. The diversity and density of these microbes ramp up from stomach to colon [2]. They participate in important physiological functions for the host and establish complex interactions with each other, ranging from mutualistic to competitive relationships, which directly or indirectly influence the host well-being [3]. A direct evidence is that the germ-free animals are more vulnerable to germs than the colonized animals [4]. In germ free animals, the mucosal cell turnover, intestinal digestive enzyme activity, local cytokine production, mucosa-associated lymphoid tissue, lamina propria cellularity, vascularity, muscle wall thickness, and motility are all lower than those in normal animals [4]. Therefore, the intestinal microbiota is supposed to produce signal factors that can regulate the function of the epithelium and sub-epithelium in the gut which is closely related with the body health.

The microbe-microbe and host-microbe dialogue is important in intestinal microbiota functioning, which has been revealed by many omics studies. *Bifidobacteria* is one of the major genera of intestinal inhabitant bacteria that make up the colon flora in mammals. In highly competitive conditions, *Bifidobacteria* applies different strategies including glycan-harvesting, glycan breakdown and cross-feeding to survive the mammal intestinal environment, resulting in changes in the microbiota composition, and a shift in the metabolism of microorganisms like the short-chain fatty acid production rate and carbohydrate utility [5]. For example, *Bifidobacteria longum* metabolizes arabinoxylan oligosaccharides into acetate that can be converted into butyrate by *Eubacterium rectale*, while *Eubacterium rectale* releases xylose that favors acetate production [6]. Predatory relationships also exist between bacteria. For example, *Bdellovibrio bacteriovorus* preys on other bacteria as the sustenance, which is conducive to regulation in the abundance and balance in bacterial communities [7,8]. Imbalance in the gut microorganisms can result in bacterial overgrowth or undergrowth, making the ecosystem vulnerable to pathogenic bacterial invasion [9]. The event of *Clostridium difficile* infection has caused up to 29,000 deaths in the USA [10]. The concomitant production of toxins from pathogenic bacteria also influences the microbiota [11] and may cause illnesses of the host [12].

## 3. Influences of Natural Polysaccharides on Intestinal Microbiota

**Figure 2:**
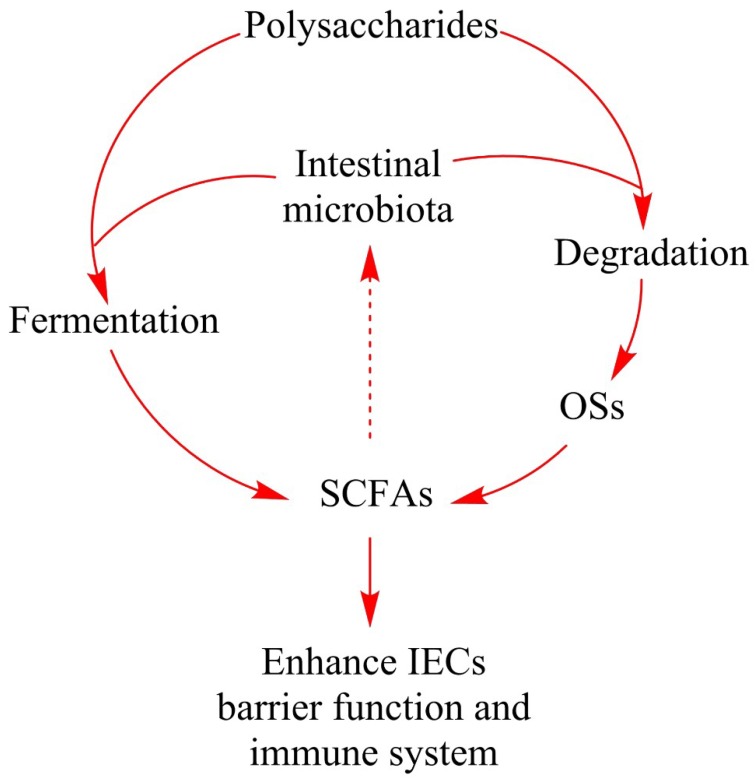
The role of natural polysaccharides in intestinal fermentation. Polysaccharides which cannot be processed by gastric and intestinal enzymes are degraded and fermented by specific intestinal microbiota. Degradation of polysaccharides produces a large number of oligosaccharides that are conducive to host health. Fermentation of polysaccharides and oligosaccharides produces SCFAs and other metabolites. SCFAs can be easily absorbed and promote the IECs barrier function and immune system. During the intestinal fermentation, polysaccharides, oligosaccharides or the metabolites like SCFAs may promote the growth of certain intestinal bacteria, thus changing the composition of intestinal microbiota and affecting the host health. Abbreviations: OSs, oligosaccharides; SCFAs, short-chain fatty acids; IECs, intestinal epithelial cells.

Polysaccharides serve as unique carbon sources for specific intestinal bacteria during fermentation. On one hand, polysaccharides are degraded by the intestinal microbiota to produce metabolites (Figure 2). On the other hand, as only certain intestinal bacteria can utilize these polysaccharides, it is necessary to investigate how these polysaccharides change and reshape the intestinal microbial community via fermentation. In a simulated human colonic fermented study, when two types of indigestible polysaccharides (apple pectin and inulin) were supplied as the energy source to three different human bowel microorganisms in vitro, two *Bacteroides* were promoted by inulin and pectin, while *Eubacterium eligens* among the *Firmicutes* was notably promoted by pectin [13]. Apple pectin has been found to increase *Firmicutes* phylum, decrease *Bacteroidetes* phylum and ameliorate the fat accumulation and body weight in diet-induced obese rats [14]. When anaerobically fermented in cecal and rectal microbial suspensions for 24 h, inulin was found to promote the population of *Lactobacilli*, *Bifidobacteria* and total bacteria, but decrease the metabolic production of skatole from l-tryptophan [15]. The exopolysaccharides of *Lactobacilli fermentum* LB-69 strain increased the growth of *Bifidobacteria* in the gastrointestinal tract [16]. Other examples include resistant starch [17,18,19], galactomannans derived from fenugreek [20], fructo-oligosaccharides [21], polysaccharides from *Barley rihane* [22] etc. Cheng et al. [23] fed the mice with different polysaccharides for 3 weeks and found that a single type of polysaccharides might increase diamine oxidase and/or trimethylamine N-oxide that were detrimental to health, but nutritionally balanced polysaccharides improved the flora diversity. Similarly, the guar gum and pectin in diet decreased the cecal diversity of *Oscillospira* and *Ruminococcaceae* in the cecum of rats [24]. Also, different polymerization degrees of dietary polysaccharides have moderately different effects on intestinal microbiota. As in a report, the low degree of polymerized inulin performed better on modulating the intestinal microbiota than the high degree of polymerized inulin did in vivo [25]. All these results reveal that the glycosidic linkage type determines the effects of the polysaccharides on the structure, diversity and metabolism of gut microbiota. 

## 4. Polysaccharide Degradation by the Intestinal Microbiota 

Polysaccharides can serve as prebiotics in our daily diet, which can promote the growth of probiotics and intestinal biodiversity [26,27]. While the human genome does not encode adequate gastrointestinal enzymes that metabolize polysaccharides, the degradation of polysaccharides needs the involvement of a series of enzymes derived from intestinal microbiota [28]. Human gut bacteria produce hundreds of polysaccharide degrading enzymes, which account for 2.62% of the total enzymes encoded by the intestinal microbiome [29]. 

Two major phyla dominate the human bowel microbiome kingdom, including the Gram-negative *Bacteroidetes* and the Gram-positive *Firmicutes*. Gram-negative *Bacteroidetes* can degrade a relatively wide range of polysaccharides, and Gram-positive *Firmicutes* tends to metabolize a series of selected polysaccharides [30]. The proportion of the *Bacteroidetes* and *Firmicutes* in the human intestines depends on our daily diets and living conditions, which have huge differences between individuals [31]. The intestinal bacteria can degrade polysaccharides via the carbohydrate active enzymes (CAZymes). The *Bacteroidetes* encodes 137.1 CAZymes per genome on average, and *Firmicutes* encodes 39.6 CAZymes per genome on average. The hydrolysis of polysaccharides happens only when they are transported to the cell surface of the bacteria. Therefore, the glycoside hydrolases and polysaccharide lyases in these bacteria must contain signal sequences for exportation to the surface of the cell. There are approximately 81% of glycoside hydrolases and polysaccharide lyases in *Bacteroidetes* that have the signal sequences, while only 19% in *Firmicutes* have the signal sequences [28]. In addition, *Bacteroides* has several carbohydrate metabolic pathways and encodes diverse degradative enzymes including glycoside hydrolases, polysaccharide lyases, and carbohydrate esterases, which confer them with the strong ability to metabolize carbohydrates [32,33]. 

**Figure 3:**
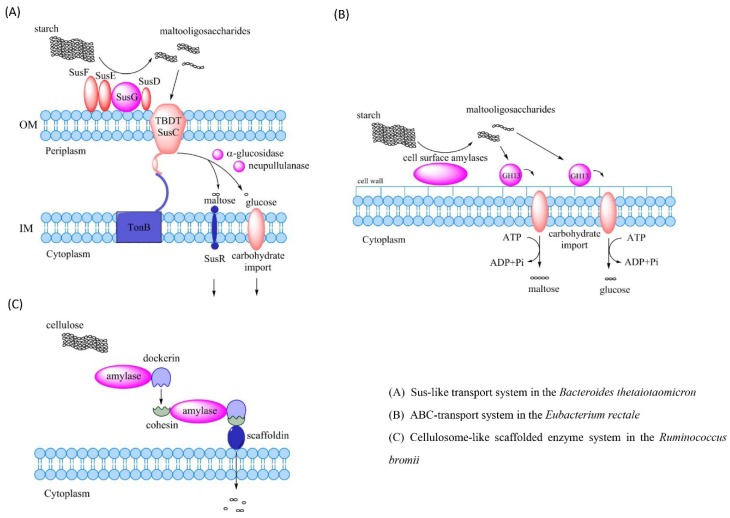
Mechanisms of the polysaccharide degradation by intestinal bacteria. (**A**) Starch utilization system (Sus) in the *Bacteroides thetaiotaomicron*, which degrades starch into maltooligosaccharides via SusG. Maltooligosaccharides are transported into periplasm by TBDT SusC through SusD, SusE and SusF and are degraded into maltose and glucose that are imported into the cytoplasm. (**B**) ABC transport system in the *Eubacterium rectale* degrades starch into maltooligosaccharides through cell surface amylases. Maltooligosaccharides are recognized by two separate ABC transport solute-binding proteins and then carried into the cytoplasm. (**C**) The cellulose-like scaffolded enzyme system in the *Ruminococcus* brings the cellulose and enzymes together on the cell surface via the dockerin-cohesion protein to degrade celluloses into monosaccharides. Abbreviations: OM, outer membrane; IM, inner membrane; TBDT, TonB-dependent transporter; GH13, glycoside hydrolase family 13; Sus, starch utilization system; ABC, ATP-binding cassette.

The mechanism of polysaccharide degradation in bacteria involves three main systems that are Sus-like transport system, ABC-transport system and cellulosome-like scaffolded enzyme system (Figure 3) [34,35,36]. The Sus-like transport system is named after the starch utilization system (Sus) [37]. The enzymes in the Sus-like transport system are encoded by the polysaccharide utilization loci (PUL) of the genome which are genetic clusters encoding essential proteins for capture, degradation, and importation of specific polysaccharides [38]. PUL has been identified in nearly all gut *Bacteroidetes* and accounts for approximately 18% of their genomes [39,40,41]. The best characterized PUL is the Sus in *Bacteroides thetaiotaomicron*. The lipoproteins SusD, SusE and SusF ignite TonB-dependent transporter SusC to transport maltooligosaccharides released by SusG into cells. These polysaccharides are degraded into maltose and glucose by α-glucosidase and neopullulanase in the periplasm, which are further transferred into the cytoplasm [39,42]. ATP-binding cassette (ABC) transport system is another polysaccharide degradation system which is common in the *Firmicutes* and *Bifidobacterium* [43,44]. ABC transport system in the *Firmicutes* degrades the long-chain starch into short-chain maltooligosaccharides via cell surface amylases. Two separate ABC transport solute-binding proteins recognize maltooligosaccharides in the length of three to seven glucose units or maltose, and carry them into the cytoplasm [45]. Cellulosome-like scaffolded enzyme system mainly targets cellulose and resistant starch. They are discovered in a cellulolytic fiber-degrading bacterium *Ruminococcus champanellensis* derived from human fecal samples [36,46]. The cellulose processing in the *Ruminococcus* is carried out via multi-enzyme complexes. These complexes are termed as cellulosomes. Cellulosomes bring substrates and enzymes together on the surface of cells through the dockerin-cohesion protein to allow the degradation of celluloses, hemicelluloses and cellulose-related polysaccharides. The dockerin-cohesion protein anchors substrates such as polypeptides to a scaffoldin protein through binding enzymes such as amylase [47,48]. The scaffoldin protein allows the functioning of carbohydrate-binding and/or cell-wall-anchoring [49]. 

## 5. Production of Short-Chain Fatty Acids during Intestinal Fermentation

Certain gut bacteria degrade polysaccharides into short-chain fatty acids (SCFAs), mainly including acetate, propionate and butyrate. SCFAs provide energy for the colon, maintain the epithelial barrier function, promote epithelial proliferation, regulate immune responses, protect against colitis and colorectal cancers and regulate certain gene expressions [50,51,52]. For example, the butyrate affects colonic health by providing energy to the epithelial cells [53]. Depending on its concentration, the butyrate can boost proliferation and differentiation of human cells and evoke apoptosis of tumor cells [54]. Several reviews have elaborated the evidences to support that low concentrations of SCFAs, particularly butyrate, can increase the risks of both colorectal cancer and inflammatory gut diseases [54,55,56]. 

Different types of polysaccharides have different impacts on the yield of SCFAs. For example, the starch fermentation by human fecal bacteria yields a higher amount of butyrate among the SCFA products than pectin fermentation. Starch incompletely digested in the small intestine is also possible to be butyrogenic [56,57]. Besides, some evidences have indicated that fructooligosaccharides are also capable of being butyrogenic [58,59]. An early study has explored the link between the supply of dietary fiber, the production of SCFAs and bowel cell proliferation, and finds that dietary fiber can stimulate gut cell proliferation [60]. 

Butyrate-producing bacteria have the ability to produce butyrate in the human colon [61]. Two of the most dominant butyrate-producing endogenous intestinal bacteria are *Faecalibacterium prausnitzii* and *Eubacterium rectale*/*Roseburia* spp. [50,61,62]. Butyrate-producing bacteria break down substrates by oxidation to obtain energy in the form of ATP. The resulting reducing equivalents are transferred to metabolic intermediates to form end products. The type of end products depends on pathways which the butyrate-producing bacteria employ. Generally, butyrate producers can also produce lactate, formate, hydrogen and carbon dioxide [63].

Butyrate-producing bacteria utilize various polysaccharides, contributing greatly to colonic fermentation of dietary carbohydrates. *Roseburia intestinalis*, one of the two major xylan degradation strains of bacteria in the human intestine, can degrade xylan by yielding high molecular mass xylanases (100–70 kDa) [64]. *Roseburia inulinivorans*, an anaerobic butyrate-producer in the human colon, utilizes glucose, starch, or inulin to produce butyrate, propionate and propanol [65,66]. However, starch utilization is more widespread. *Roseburia* releases a type of extracellular amylase to degrade starch on the cell surface via a sortase-mediated mechanism [45]. On the other hand, l-sorbose and xylitol, as prebiotic stimulus, can promote the growth and metabolic activity of butyrate-producing *Anaerostipes* spp. in vitro [67]. According to Ravn et al. [68], oligosaccharides from wheat bran can promote butyrate production by the butyrate-producing bacterial genera *Faecalibacterium* and *Intestinimonas*. Moreover, dietary fibers in diets can modulate the abundance and activity of butyrate-producing bacteria in the large intestine [69]. However, not all natural polysaccharides contribute to butyrate production. For example, a study reported that the butyrate-producing bacteria decreased when the adult volunteers were treated with fructooligosaccharides and galactooligosaccharides for 14 days (16 g/day) [70].

The population of the colonic butyrate-producing bacteria is closely related to host health. From studying the microbiota in the stools collected from hundreds of diabetic patients, it was found that only a moderate dysbiosis occurred in the diabetic population, whereas a decline in butyrate-producing bacteria and an increase of opportunistic pathogens were observed [71]. Interestingly, a similar phenomenon was observed in human colorectal cancer patients [72] and aging people [73]. There is a large number of studies reporting that the butyrate-producing bacteria are depleted in inflammatory bowel diseases (IBD) patients [74,75,76]. For example, *Clostridium coccoides* in fecal samples or on the gut mucosa of ulcerative colitis patients were reduced [77]. Even in the colonic mucosa of HIV patients, abundance of *Roseburia intestinalis* had a relatively low level [78]. Some even propose that certain butyrate-producing bacteria like *Butyricicoccus pullicaecorum* may serve as an available therapeutic tool for inflammatory bowel diseases [79]. Nylund et al. [80] found that the severity of atopic diseases was closely correlated with low abundance of butyrate-producing bacteria in the human intestine. In addition, supplementation with *Clostridium butyricum* enhanced expressions of the peroxisome proliferator-activated receptor, insulin signaling molecules and mitochondrial function markers in diabetic mice [81]. Some investigators have introduced new concepts, like ‘*Clostridia*-directed enzyme prodrugtherapy’ and ‘Combined bacteriolytic therapy’, combined with immune modulation for tumor therapy. It is suggested to treat metastases at early stage with the genetically engineered *Clostridia* which can induce phagocytosis and humoral immune response to avoid invasion of tumor cells [82]. Similarly, Minton et al. [83] intravenously injected *Clostridial* spores to permeate and selectively germinate in the hypoxic areas of the tumor. Ohkawara et al. [84] fed the mice with a new strain of butyrate-producing *Butyrivibrio fibrisolvens* (MDT-1) at a dose of 10^9^ cfu for 4 weeks and found that the number of colorectal aberrant crypt foci, putative preneoplastic lesions and aberrant crypts were reduced. However, there was not the same result in the MDT-1 cell homogenate. The activity of β-glucuronidase decreased, the NK and NKT cells and butyrate production increased, which indicated that MDT-1 could decrease the formation of aberrant crypt foci in the colon and rectum of mice.

## 6. Polysaccharides and Health

**Figure 4:**
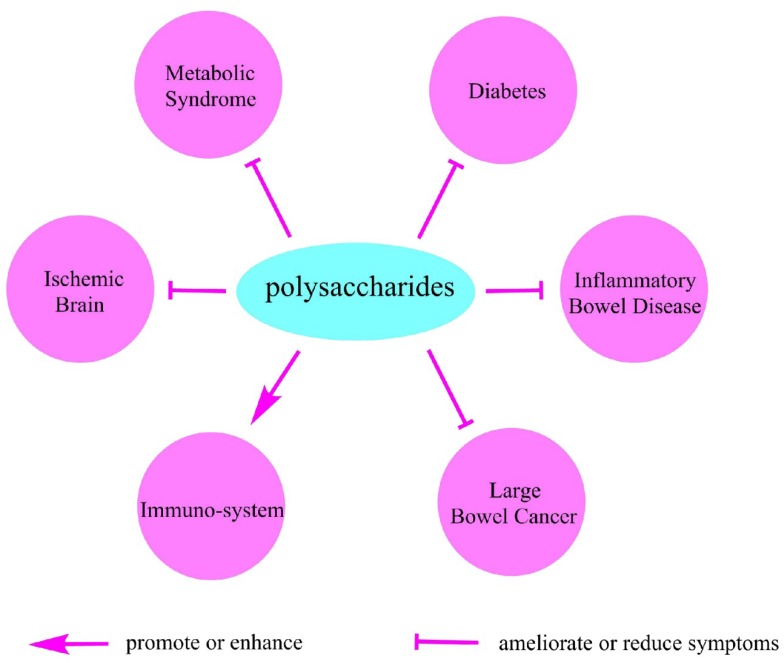
The beneficial effects of polysaccharides on health.

Because most polysaccharides cannot be completely digested by our alimentary system, the beneficial effect of many polysaccharides is mainly dependent on its fermentability as well as physiochemical properties including water-holding capacity and bile acid binding ability. Therefore, the natural polysaccharides benefit our health mainly by slowing gastric emptying [85], physically improving the bowel function [86], modulating the gut microbe structure [87], working as substrates for microbial fermentation [85], and protecting the immune system [88,89]. Here we listed several health benefits that are closely associated with different natural polysaccharides (Figure 4).

### 6.1. Metabolic Syndrome

Metabolic syndrome is a clustering of medical metabolic conditions including obesity, high blood pressure, high blood glucose, high triglycerides and low high-density lipoprotein levels. Metabolic syndrome is frequently reported to be associated with deranged microbiota. Antibiotic-induced changes of gut microbiota can reduce metabolic endotoxemia and cecal lipopolysaccharide so as to improve metabolic parameters in both high-fat-fed and ob/ob mice [90]. Many polysaccharides have been reported to effectively ameliorate metabolic syndrome. For example, the apple-derived pectin was found to reduce body weight gain and the excessive accumulation of fat in diet-induced obese mice [70]. Soluble dietary fiber suppressed weight gain and fat accumulation by increasing energy expenditure and modulating gut microbiota [91]. In addition, exopolysaccharides isolated from Kefir grains decreased body weight gain, adipose tissue weight, and plasma very-low-density lipoprotein cholesterol concentration of high-fat diet-fed mice [92]. However, some polysaccharides only regulate the composition of gut microbiota but are not conducive to obesity prevention. For example, in the bowel of rats consuming whole-grain flour with a high content of resistant starch, the proportion of *Firmicutes* phyla and the *Lactobacillus* genus increased but no difference in abdominal fat accumulation was observed [93]. Taken together, there is not sufficient evidence to verify that all polysaccharides have potential to treat obesity by regulating gastrointestinal microbiota, though many polysaccharides have shown different effects on modifying the diversity and abundance of intestinal flora. 

Increased energy intake is a key factor that leads to obesity. Calorie dense diets promote increased caloric intake [94,95]. Indigestible polysaccharides have a short-term control of food consumption both within and between meals [96]. Due to the bulking effect of many natural polysaccharides, the food intake or energy intake will be reduced accordingly [97]. The satisfaction of appetite that develops during the course of indigestible polysaccharide intake can help stop further food intake. As the gastrointestinal tract (GI) has a close connection with the brain, the full status from the GI tract can be mechanically sensed to signal the brain for food control [98].

Higher dietary fiber intake improves glucose metabolism and predicts higher glucose control [99,100,101,102], especially viscous fiber [103,104]. Viscous fiber consumption can slow down glucose absorption to avoid quick peaking of blood glucose. The high carbohydrate diet accompanied with a high fiber diet can improve blood glucose control and reduce plasma cholesterol levels in diabetic patients without increasing plasma insulin and triglyceride concentrations [105]. This is not only beneficial for diabetic patients but can also protect healthy people against metabolic syndrome. 

Indigestible polysaccharides can reduce fat absorption partly by binding to fat molecules and increasing their excretion [106]. Dietary fiber can bind to bile acids [107] or bile salt [108]. Bile acid is critical for the formulation of micelles and solubilize lipids. The reduction of bile acid activity is supposed to directly lower fat absorption. Acid soluble polysaccharides from *Dioscorea opposita* Thunb showed strong hypoglycemic activity at high doses (400 mg/kg). The results indicated that the polysaccharides promoted antioxidant enzyme activity and stimulated glucose disposal of alloxan-induced diabetic rats [109]. The polysaccharides isolated from *Enteromorpha* also showed the ability of lowering blood lipid and antioxidant activity in vivo [110]. Polysaccharides extracted from *Momordica charantia* lowered blood lipids and increased the activity of superoxide dismutase, catalase and non-protein sulfhydryls and reduced the level of lipid peroxidation in rats [111]. Many polysaccharides from different resources have similar effects. Korean mulberry fruits Oddi polysaccharides decreased the number of fat cells through inducing mitochondrial dysfunction and apoptosis in pre-adipocyte cells, and prevented obesity [112]. *Cymodocea nodosa* sulphated polysaccharides reduced total cholesterol, triglycerides and low-density lipoprotein cholesterol, and increased the levels of high-density lipoprotein cholesterol. A decrease in the body weight and a suppression in lipase activity of obese rats in serum and intestine was observed [113]. Codium fragile sulphated polysaccharides showed the similar effects in serum, and ameliorated hyperlipidemia of induced obese rats [114]. Chinese Liupao tea polysaccharides reduced body weight and cholesterols of hyperlipidemic rats [115]. *Ophiopogon japonicus* polysaccharides promoted weight loss and decreased the mass of adipose tissue in the obese mice by increasing energy expenditure [116]. In addition, the polysaccharides from *Ophiopogon japonicus* gathered bile acids and decreased their reabsorption in the bowel to promote cholesterol catabolism [117].

### 6.2. Diabetes

Diabetes is one of the current leading causes of death. The diabetic population has been increasing at a stable rate since 2000 and is predicted to reach 4.4% worldwide in 2030 [118]. Diabetes is associated with a wide variety of complications. Some severe complications include retinopathy, nephropathy, neuropathy, coronary heart disease, hypertension, peripheral vascular disease and amputations [119]. Dietary modification plays an essential role in diabetes management, typically type 2 diabetes [120,121,122]. For example, the diet incorporated with cereal fiber was effective in prevention of diabetes [123,124] and a high intake of cereal fiber for diabetic patients also improved their health conditions [125]. 

Consumption of fiber like polysaccharides has been frequently indicated to protect against the incidence of diabetes [126,127,128,129]. In contrast, diabetic patients usually have a lower dietary fiber intake [130,131]. Diabetic patients have changed gut microbiota compared with non-diabetic people [71,132]. There are some investigations about polysaccharides in diabetes therapies. Polysaccharides can affect the progression of diabetes via modifying gut barrier and microbiota homeostasis. A western diet combined with resistant starch was supplied to germ-free mice or mice containing microbiota. The insulin sensitivity was improved in resistant starch-fed normal mice, and the insulin levels were also improved in resistant starch-fed germ-free mice. Gene expressions of adipose tissue macrophage markers and cecal concentrations of several bile acids were reduced in both germ-free and normal mice [133]. According to Zhang et al. [134], in the inulin-treated diabetic rat groups, the abundance of probiotic *Lactobacillus*, *Lachnospiraceae*, *Phascolarctobacterium* and *Bacteroides* which produced SCFAs significantly increased, while the abundance of lipopolysaccharide-producing *Desulfovibrio* reduced. Exopolysaccharides separated from the fermentation liquor of *Hypsizigus marmoreus* ameliorated the histopathological alterations in the kidney of streptozocin-induced diabetic mice. Additionally, an increase in superoxide dismutase (SOD), glutathione peroxidase (GPx), catalase, total antioxidant capacity, and albumin, and a decrease in the contents of malondialdehyde, lipid peroxide and levels of serum urea nitrogen and creatinine were observed [135]. Additionally, the ratio of *Firmicutes*/*Bacteroidetes* and the richness of *Ruminococcaceae* and *Lactobacilli* increased to achieve antidiabetogenic effect. Liu et al. [136] fed type 2 diabetes rats with *Cordyceps sinensis* polysaccharides for 4 weeks. The insulin sensitivity index was increased, the levels of fasting blood glucose and fasting insulin were reduced, and the number of apoptotic cells and the expressions of both homologous protein and c-Jun were decreased in the diabetic rats. *Cordyceps cicadae* crude polysaccharides decreased blood glucose of diabetic rats, total cholesterols, triglycerides, low-density lipoprotein, malondialdehyde, urea, creatinine, alanine transaminase, aspartate aminotransferase and alkaline phosphate, and increased body weight, high-density lipoprotein, SOD and GPx [137]. According to Tang et al. [138], six fractions of polysaccharides derived from different parts (whole plants, roots and leaves) of *Anoectochilus roxburghii* and *Anoectochilus formosanus* were fed to streptozocin-induced diabetic mice and the body weight, blood glucose, glycogen, insulin, total cholesterols, triglycerides, low-density lipoprotein, high-density lipoprotein, malondiadehyde, and antioxidant enzyme activities in the liver and kidney of mice were tested. They found that all polysaccharides had antidiabetic activities, and root polysaccharides performed better than leaf polysaccharides in the antidiabetic activities.

Gastrointestinal symptoms are common in diabetic patients with possible disordered neuroendocrine functions [139]. Many undigested polysaccharides can be excreted whereas a portion can be fermented by gut bacteria. The ‘in and out’ process allows these polysaccharides to have opportunities to carry part of gut bacteria, dead cell debris as well as toxins to be removed together. Similarly, polysaccharides can also reduce nutrient absorption evidenced by increased fecal output when incorporating dietary fiber into diet [140]. Soluble dietary fiber has been applied as a treatment for slowing transit constipation by regulating intestinal microecology. Clinical improvement and remission of constipated patients were observed, and the patients felt satisfied with improved gastrointestinal quality-of-life index with continuous consumption of soluble dietary fiber for 4 weeks [141]. 

There are researches demonstrating the potential beneficial effects of dietary fiber in the chronic kidney disease (CKD) population by reducing the serum urea and creatinine levels [142]. Gum arabic was supplemented to CKD patients at 10–40 g/day, and significantly decreased serum sodium level and C-reactive protein level, which was effective to alleviate these patients’ morbidity and mortality [143]. Oral administration of fucoidan derivatives from *Laminaria japonica* significantly decreased the serum urea nitrogen and serum creatinine levels of CKD rats, ameliorating the histopathological symptom of renal tubules, interstitium and mesangial areas via substituting the electronegative element of the glomerular cells and suppressing mesangial cell proliferation [144]. In addition, two sulfated polysaccharides of low molecular weight fucoidan and high uronic acid fucoidan deprived from *Laminaria japonica* Aresch showed the same effect on CKD rats. Both reduced the peroxidative and renal damage and ameliorated CKD [145].

### 6.3. Inflammatory Bowel Disease

Inflammatory bowel disease (IBD) is a type of intermittent inflammation occurring in the gastrointestinal tract [146], including ulcerative colitis and Crohn’s disease, and its incidence has escalated for the past few years [147,148]. The clinical symptoms of IBD include persistent diarrhea, emesis, hemafecia, weight loss and ache. Many drugs used for IBD therapy in clinic have been reported with adverse effects, such as antibiotics, aminosalicylates and corticosteroids [149,150]. Yue et al. [151] fed the colitis rats with wild *Jujube sarcocarp* polysaccharides to investigate the protective effect against IBD. The results showed that the polysaccharides ameliorated the inflammatory response through lowering TNF-α, IL-1β, IL-6 and MPO activity and increased AMPK activity in Caco-2 cell, stimulated by TNF-α in colitis rats. Similarly, the supplementation of a guar gum and partially hydrolyzed guar gum mixture remarkably reduced the clinical score of dextran sulfate-induced colitis in rats [152]. After the oral administrations of *Lentinus edodes* β-glucan in mice, body weight was increased, disease activity index was decreased and inflammatory symptoms were alleviated [153]. Similarly, Segarra et al. used 27 IBD dogs treated by chondroitin sulfates and several prebiotics including resistant starch, β-glucan, and mannaoligosaccharides for 6 months and found that the canine IBD activity index score was decreased [154].

### 6.4. Large Bowel Cancer

Diets rich in natural polysaccharides, especially dietary fiber, can protect against development of colorectal cancer. Hu et al. [155] fed colitis-associated colorectal cancer rats with diets containing 10% resistant starch for 2 weeks. They found that colitis-associated colorectal tumor multiplicity and adenocarcinoma formation significantly decreased in the dietary resistant starch group. Panebianco et al. [156] fed xenograft pancreatic cancer mice with diets containing resistant starch, discovering that the growth and proliferation of pancreatic tumors were significantly retarded. Additionally, inulin suppressed colonic tumorigenesis related with changes in the cecal microbial flora [157]. Dietary fiber can also promote the growth of probiotic bacteria and prevent carcinogenesis of colorectal cancer [158]. By contrast, lack of dietary fiber harms the health of the intestine. A dietary fiber-deprived diet can weaken the colonic mucus barrier, as a result, the mucosal pathogen *Citrobacter rodentium* has a greater chance of accessing to the epithelium that increases the risk of lethal colitis [159]. Exopolysaccharides from *Parachlorella kessleri* inhibited the proliferation of colon carcinoma cells (CT26) through suppressing cell growth directly and inducing the host antitumor immune responses [160]. Exopolysaccharides of *Lactobacillus casei* SB27 isolated from Chinese yak milk significantly inhibited the growth of colorectal cancer cells (HT-29) and enhanced the expressions of Bad, Bax, Caspase-3 and -8 genes to induce apoptosis [161].

In addition to gut-related cancers, polysaccharides may also help prevent other types of cancers. For example, the polysaccharides from *Sargassum fusiform* significantly inhibited the growth of nasopharyngeal carcinoma cell line (CNE) by increasing serum cytokines and IgM levels in CNE-bearing mice and stimulated the secretion of cytokines from peritoneal macrophages, which stimulated the proliferation of splenic lymphocytes and increased the expression of IgM in splenic lymphocytes [162]. Meng et al. [163] showed that the anti-tumor effect of *Letinous edodes* polysaccharides was achieved by stimulating T cells and other immune cells. These cells can trigger a variety of reactions such as the expression of certain cytokines. Mulberry polysaccharides have an obvious anticancer effect on cancer cells [164]. The mechanism of polysaccharides in anti-cancer activity is not clearly understood, but the polysaccharides in regulating the immune system via intestinal fermentation is probably involved. 

### 6.5. Polysaccharides Regulate Immuno-System 

Since immunosuppression often occurs with diabetes [165,166,167], diabetic patients usually suffer from infections from different pathogens [168]. Immunosuppression is associated with an impaired inflammatory response [169,170]. Different polysaccharides modulate the intestinal immune system in different ways. Specifically, many immune indices were shown to be affected by different polysaccharides including serum IgE, IgA, IgG, IgM, CD4^+^ T-cells, CD4^+^/CD8^+^ ratio, mesenteric lymph node lymphocytes, cecal densities of CD8^+^ intraepithelial lymphocytes and CD161^+^ natural killer cells [88,171]. Sweet cherry pectic polysaccharides stimulated the NO release from macrophage-like cells and the expression of several immune-related molecules including TNF-α, interleukins (IL-6 and IL-1β), granulocyte colony stimulating factor, inducible nitric oxide synthase and cyclooxygenase-2 [172]. *Diaphragma juglandis fructusa* polysaccharides showed a potential antitumor and immunomodulatory activity. The polysaccharides remarkably inhibited proliferation of HepG2 and BGC-82 cells, promoted phagocytosis, and increased the release of NO, TNF-α, IL-6, IL-10, and the corresponding mRNA expression [173]. Jia et al. [174] found that polysaccharides of *Rhynchosia minima* root increased the proportion of CD3^+^ and CD4^+^ T lymphocytes, the CD4^+^/CD8^+^ ratio of splenocytes, improved the phagocytic ability of macrophages, increased the production of NO and the secretion of cytokines (TNF-α, IL-6, and MCP-1) from macrophages, and decreased cyclophosphamide-induced immunosuppression in mice. Exopolysaccharides of *Lactobacillus delbrueckii* OLL1073R-1 activated porcine intestinal epithelial cells (PIE cells), triggered innate immune response and increased the expression of IFN-α and IFN-β in PIE cells as well as the expression of the antiviral factors MxA and RNase L [175]. Exopolysaccharides of *Auricularia auricula-judae* increased the release of NO and cytokines (IL-6, IL-10 and TNF-α) in Raw 264.7 cells (mouse leukaemic monocyte macrophage cell line) [176].

The diet that facilitates symbiosis can improve the immune system through anti-inflammatory and/or immune modulatory substances like SCFAs, whereas the diet that induces dysbiosis can induce immune dysregulation [53]. Apparently, indigestible polysaccharides can improve the immune system by modulating the gut microbiota [129]. A recent report showed that fermentable dietary fiber elevated the innate immune response and disease resistance partly by upregulating immune-related gene expressions [177]. Dietary polysaccharides can promote the proliferation of intestinal epithelial cells and activate intestinal immune cells. *Bupleurum chinense* is a famous Chinese medicine with thousands of years of history. Consumption of *Bupleurum* polysaccharides increased the proportion of dendritic cells in bone marrow and liver of septic mice [178]. Guo et al. [179] found that the treatment of exopolysaccharides from *Lactococcus lactis* subsp. *lactis* enhanced macrophage phagocytosis, spleen and thymus indices and hemolytic complement activity. One of the mechanisms is the generation of the bioactive molecules from intestinal fermentation. For example, a soluble peptidoglycan released by the gut bacteria can translocate into the circulation for the remote systematic priming of neutrophils in the bone marrow [180]. Another reason is that polysaccharides can produce the immunomodulatory products from the gut microbiota particularly SCFAs [53].

### 6.6. Ischemic Brain

It is not surprising that the polysaccharides can affect the brain health via the identified gut-brain axis [181,182,183]. The polysaccharides from *Gastrodia elata* Blume improved the conditions of the focal cerebral ischemia rats after 2 weeks’ consumption. The expressions of brain-derived neurotrophic factors and stem cell factor proteins in the caudate putamen increased significantly [184]. According to Su et al. [185], *Lonicera japonica* flower polysaccharides exerted a neuroprotective effect through anti-oxidant activity on focal ischemia/reperfusion injury in the rat brain. Another study showed that *Lycium barbarum* derived from polysaccharides ameliorated hyperglycemia-aggravated ischemic/reperfusion brain injury through equilibrating mitochondrial fission and fusion. An increase in phospho-Drp1 and a decrease in Opa1 that negatively correlated with LBP dosage were observed [186]. In addition, according to Shi et al. [187], *Lycium barbarum* polysaccharides protected against ischemic injury via modulating NR2B and NR2A signaling pathways. 

## 7. Conclusions

The gut microbiota is regarded as an essential dynamic organ that functions in nourishment, epithelial development and innate immunity [188]. One important benefit of the dietary polysaccharides to human health is due to its fermentability in gut [87]. It’s been known quite well that dietary fiber is able of impacting fecal microbiota [189,190]. Fermented products from these polysaccharides, especially SCFAs such as propionate, are bioactive molecules with health benefits [191]. It’s proposed that the dietary polysaccharide-deriving SCFAs could be converted into glucose and/or directly signal intestinal receptors and therefore contribute the benefits via gut-brain neural circuits [181,182,183]. Moreover, fermented polysaccharides can facilitate the beneficial bacteria to generate bioactive molecules important for the normal maturation of the host immune system [192,193]. Several questions on how the dietary polysaccharides interact with the gut system need to be deeply studied. For example, since the differences exist between the stool and adherent mucosal communities [188], current human researches on the adherent mucosal communities may be more valuable. With rapid development of metagenomic and other omics techniques, how different polysaccharides affect the gut microbiota can be further evaluated.
